# Outcomes of Mutual Support Groups on Well‐Being, Academic Skills, Career Confidence, and Psychological Support Attitudes Among Higher Education Students

**DOI:** 10.1002/pchj.70053

**Published:** 2025-09-29

**Authors:** Zamira Hyseni Duraku, Liridona Jemini Gashi, Artë Blakaj, Viola Greiçevci, Vali Ibrahimi, Fisnik Eger, Donarta Uka, Rrezarta Vllasaliu, Adea Dobra, Rajma Brenoli

**Affiliations:** ^1^ Department of Psychology Faculty of Philosophy, University of Prishtina Prishtinë Kosovo

**Keywords:** career confidence, intention to seek help, mutual support groups, social support, student well‐being, study skills

## Abstract

Mutual support groups are increasingly implemented in higher education settings across high‐income countries to promote peer‐based support, with demonstrated benefits for emotional well‐being and social connectedness. However, their impact on other domains of students' lives remains underexplored, particularly in low‐ and middle‐income contexts. This study investigates the outcomes of mutual support groups by examining students' perceived changes in mental well‐being, academic skills, career certainty, social support attitudes, interpersonal functioning, and attitudes toward seeking psychological help. Using a qualitative retrospective approach, open‐ended responses were collected from 20 Kosovar students (aged 18–25 years) at a major public university after a 5‐week support group program. Data were thematically analyzed using a deductive approach based on predefined themes aligned with the study's objectives. Findings revealed that participating in mutual support groups contributed to reduced stress, lower anxiety, and improved mood, as students felt heard and emotionally supported by peers facing similar challenges. Students adopted more effective study habits and time management techniques through the sharing of practical strategies and encouragement. Open discussions about career uncertainty fostered clarity and confidence in students' academic and professional goals. Hearing from the perspectives of others on mental health reduced internalized stigma and increased willingness to seek psychological support. The group setting also enabled students to develop stronger interpersonal skills, including empathy, emotional expression, and a sense of connection and belonging. This study highlights the potential of mutual support groups as effective peer‐led supplements in higher education by emphasizing improvements in student well‐being, academic development, and mental health attitudes.

## 1 Background

High levels of stress and anxiety among higher education students are consistently reported globally—from the United States to European countries, including the United Kingdom (Allen et al. 2022; Auerbach et al. 2016; Długosz 2022)—as well as in Southeastern Europe, particularly Kosovo (Arënliu et al. 2021; Hyseni Duraku et al. 2023). This period is often marked by a combination of interconnected personal, academic, and transitional stressors (Hyseni Duraku, Davis, Arënliu, et al. 2024), which, when compounded by limited access to psychological services and weak institutional support structures, can undermine mental health, reduce academic performance, and hinder long‐term personal and professional development (Hyseni Duraku and Hoxha 2018; Jemini‐Gashi and Hoxha 2024).

A core academic challenge lies in students' inadequate preparation for university demands. Many individuals struggle with underdeveloped study skills and difficulty adapting to new expectations, particularly during the transition from secondary to higher education (Auerbach et al. 2016; Mohammadyari 2012). These issues often reflect a lack of holistic, skills‐based learning approaches at earlier stages, resulting in heightened academic pressure, test anxiety, and lower achievement—all of which undermine student motivation and performance (Alrashidi et al. 2016; Hu and McCormick 2012; González and Paoloni 2015). In Kosovo, these gaps are particularly acute, with students reporting challenges with learning strategies and high academic anxiety (Hyseni Duraku 2017; Hyseni Duraku 2021, 2023; Hyseni Duraku and Hoxha 2018).

Compounding academic stress is the growing uncertainty about future careers. Many students feel unprepared to make informed decisions or navigate employment opportunities (Salim et al. 2023). In Kosovo, these challenges are intensified by the absence of institutionalized career guidance and limited coordinated support from schools and families (Jemini‐Gashi and Kadriu 2022; Jemini‐Gashi and Hoxha 2024).

Simultaneously, stigma and cultural prejudice around mental health continue to restrict awareness and help‐seeking among students (Corrigan and Rüsch 2002), especially in low‐ and middle‐income contexts (WHO 2022). In Kosovo, Albania, and North Macedonia, such barriers are amplified by familial norms that discourage open discussions of mental health—particularly among Albanian youth (Hyseni Duraku, Davis, Blakaj, et al. 2024). The lack of accessible, youth‐centered psychological services exacerbates this issue. In Kosovo, counseling remains mostly privatized and financially inaccessible for many students. Meanwhile, public services follow a medicalized model that rarely addresses students' preventive or developmental needs (Hyseni Duraku, Davis, Arënliu, et al. 2024). While some private universities in Kosovo offer university‐based counseling services and related psychological support programs, these services are still in the early stages of development across the public higher education system. This system is primarily organized through a network of state‐funded institutions, with the largest university located in the capital, Prishtina. Although recent reforms have increasingly emphasized student‐centered learning, and national accreditation frameworks call for the integration of emotional and academic support services, structured programs remain limited in their implementation. Many current initiatives remain pilot efforts, often led voluntarily by academic departments rather than being embedded institution‐wide.

Social support is also widely recognized as a crucial factor influencing students' academic engagement and emotional well‐being. This becomes particularly important during the transition to higher education, when students must adapt to unfamiliar environments and build new relationships (Chang et al. 2020). Strong peer and faculty connections enhance students' sense of belonging—an essential driver of motivation, persistence, and academic success (Axelson and Flick 2010; Chen et al. 2023; Kuh 2003). In Kosovo, informal peer relationships have been found to play a significant role in reducing academic anxiety and promoting help‐seeking among university students (Hyseni Duraku et al. 2023). Similarly, a study with high school students reported that more than half received meaningful emotional and practical support from classmates and friends, which helped them cope with academic pressures and make clearer career decisions (Jemini‐Gashi and Hoxha 2024). Despite these benefits, many students struggle to form social connections at university. Large class sizes, limited extracurricular opportunities, and a lack of structured peer interaction spaces often restrict relationship‐building (Eames and Stewart 2008). Socioeconomic status and social identity factors can further constrain students' ability to engage with campus life and access support networks (Nieuwenhuis et al. 2019).

In response, peer‐to‐peer mutual support groups have emerged as a promising strategy to foster connection and enhance student well‐being. Initially developed in healthcare settings (Darby Penney 2018), mutual support groups are now increasingly applied in education. These voluntary, self‐organized groups bring together individuals facing similar challenges to share experiences and offer reciprocal support (Steinberg 2014). In higher education, such groups serve as platforms for students to exchange academic strategies, explore career concerns, and provide emotional support and role modeling—contributing to improved performance, reduced anxiety, and greater confidence in academic and professional paths (Crisp et al. 2020; Salim et al. 2023).

Recent evidence reinforces these benefits. A 6‐week study in the United Kingdom found that students experiencing emotional distress were more likely to join mutual support groups, with regular participation linked to improved well‐being (Pointon‐Haas et al. 2024). Additional outcomes include smoother transitions to university life, stronger social ties, enhanced self‐esteem, better communication, and a greater sense of belonging (Byrom 2018; Casiday et al. 2008; Collings et al. 2014; Dearlove et al. 2007; Oarga et al. 2015). During the COVID‐19 pandemic, the implementation of peer‐led mutual support groups among psychology students at a public university in Kosovo also demonstrated positive outcomes. These groups provided safe and inclusive spaces for sharing experiences, building trust, and developing coping strategies, while also enhancing students' communication skills, confidence, and ability to apply academic knowledge in practice (Hyseni Duraku et al. 2025).

Several theoretical perspectives can further help explain the mechanisms and potential benefits of mutual support groups. To understand the group dynamics within mutual support settings, this study draws on resource theory and social exchange theory. The resource theory (Foa and Foa 1974, 1980) categorizes exchange into six types: love, status, information, money, goods, and services. Love, status, and information were relevant in the mutual support groups. Love is exchanged through warmth, comfort, and empathy, creating strong emotional bonds and supportive group environments (Foa and Foa 1980). Status occurs when members share valuable information and receive positive recognition, thereby enhancing their social standing within the group (Foa and Foa 1974, 1980). Information, especially experiential knowledge, is crucial, as participants share personal experiences and coping strategies, leading to validation, emotional release, and reduced social isolation (Coates and Winston 1983; Helgeson and Gottlieb 2000).

Social exchange theory, as articulated by sociologists such as George Homans and Peter Blau, posits that social behavior is driven by an exchange process in which individuals seek to maximize benefits and minimize costs in their interactions (Stafford 2017). This framework can help explain the reciprocal nature of interactions within mutually supportive groups. In these groups, the benefits—ranging from emotional support to the sharing of practical knowledge—may contribute to participants' improved well‐being. Simultaneously, the primary costs involve the time and emotional energy invested in participation.

To understand how such interactions may influence students' attitudes and intentions, this study draws on the elaboration likelihood model (ELM) (Petty and Cacioppo 1986) and the theory of planned behavior (TPB) (Ajzen 1991). ELM posits that attitude change occurs through two routes: the central route, involving thoughtful reflection on message content, and the peripheral route, driven by surface‐level cues such as peer likability or emotional tone. In mutual support groups, personal sharing and meaningful discussion may encourage central processing, while peer relatability may foster peripheral influence.

Complementing this, TPB explains how behavioral intentions—such as seeking psychological support—are shaped by three socio‐cognitive factors: attitudes toward the behavior, subjective norms (i.e., perceived social pressure), and perceived behavioral control (i.e., belief in one's ability to perform the behavior). Participation in a mutual support group may positively influence all three domains: shaping attitudes by normalizing open discussion of mental health, reinforcing subjective norms through peer validation, and enhancing perceived control by building a sense of self‐efficacy. Together, these mechanisms may increase students' willingness to seek help when needed and reduce psychological barriers such as internalized stigma or uncertainty about accessing support.

Therefore, drawing on these theoretical frameworks and previous research on peer‐led mutual support groups, this study aims to contribute to the expanding evidence base by examining a range of perceived outcomes among higher education students from various disciplines of study, in an under‐researched context and across domains of student experience that remain underexplored. Specifically, the study examined the outcomes of participation in mutual support groups on various aspects of students' experiences at a major public university in Kosovo. These include their emotional well‐being, academic anxiety, career‐related confidence, attitudes toward seeking psychological support, and sense of social connection.

The insights from this study may be valuable in informing the development of preventive and peer‐based support models within higher education. This is particularly relevant in low‐resource contexts. In particular, where professional mental health services are limited, inaccessible, or underutilized, peer support networks can serve as a complementary approach to bridging the gap between student needs and available services (Hyseni Duraku et al. 2025). The findings presented here align with the growing recognition of higher education institutions' responsibility to protect and promote student mental health. They may support advocacy efforts in countries where student‐centered mental health systems are still emerging and where national higher education policies have yet to include explicit objectives for student well‐being (Galán‐Muros et al. 2024).

## 2 Methods

### 2.1 Study Design

This study used a retrospective qualitative design to explore the perceived psychological, academic, interpersonal, and attitudinal outcomes of participating in peer‐led mutual support groups. Data were collected at a single point after the 5‐week intervention. Participants were asked to evaluate their well‐being, academic engagement, social interactions, and attitudes both before and after attending the mutual group meetings. This approach enabled students to assess change through a consistent internal frame of reference, thereby minimizing response‐shift bias, which occurs when participants' understanding of concepts such as stress or support evolves (Howard et al. 1979). Furthermore, a retrospective design was chosen for its practicality in early‐phase, low‐resource support programs and its suitability for culturally sensitive topics such as mental health. Post‐only designs reduce participant burden and may enhance an open reflection, particularly in contexts where pre‐intervention surveys limit engagement (Geldhof et al. 2018). In addition, administering a single post‐program evaluation can conserve time and resources, allowing staff to prioritize delivery and build rapport before addressing potentially sensitive issues with participants (Pratt et al. 2000).

### 2.2 Participants

The study included 20 participants aged 18–25 years (*M* = 21.14, SD = 2.47) from a major public university in Kosovo. Sixty percent were undergraduate students—30% in their first year, 15% in second, and 15% in third—while the remaining 40% were master's students, evenly split between first and second year. Participants represented a diverse range of academic disciplines, including psychology, law, medicine, and economics. The sample was predominantly female (80%), and most participants reported residing in urban areas. Students came from five of Kosovo's seven regions. Most (85%) had no prior experience with psychological services or any emotional support within their educational institutions, and only two reported using mental health apps such as *Calm* or *Headspace*.

### 2.3 Materials and Procedures

#### 2.3.1 Intervention

The mutual support groups were implemented during the Spring 2024 semester as part of the “Students for Students” initiative in the Department of Psychology, facilitated by student and alumni volunteers from the same department. These facilitators had previously participated in similar groups during the COVID‐19 period and had received training in group facilitation and ethical principles from academic mentors within the department. The program was further supported by a facilitation manual, outlining group objectives and discussion guidelines.

Invitations to participate were shared publicly through the department's social media platforms for its support programs. The groups were open to all students enrolled at the major public university in Kosovo. Participants from all academic units, regardless of their academic level or field of study, were eligible, provided they were actively enrolled during the Spring 2024 semester. Interested individuals registered via an online sign‐up form. They were randomly assigned to one of six groups, each comprised of 10–12 participants and led by one facilitator and one co‐facilitator. Sessions were held once per week over 5 consecutive weeks. Discussion topics addressing academic, emotional, and interpersonal concerns relevant to participants' experiences were co‐developed and addressed with participants. Participation was entirely voluntary, and students were free to withdraw at any time.

#### 2.3.2 Data Collection

Participation in the evaluation was strictly voluntary. During the first group session, facilitators informed students that a post‐intervention review would take place at the conclusion of the 5‐week program. A link to a sign‐up form was shared with students interested in participating. The form collected email addresses and served as a record of informed consent to receive the post‐program questionnaire. After the final (fifth) session, a survey invitation was administered via Google Forms and distributed through the official email address of the Department of Psychology Support Program. The survey remained open for 2 weeks, and a reminder email was sent midway through the period. Of the 56 students who participated in the mutual support groups, 40 provided informed consent to be contacted for the evaluation. Of these, 35 submitted responses. However, only 20 participants met the inclusion criterion. To be included in the final sample, students had to (1) provide informed consent, (2) complete the full questionnaire, and (3) have attended all five mutual support group sessions. Participants who missed one or more sessions or failed to complete the questionnaire were excluded to ensure that the evaluation accurately reflected the experiences of those fully engaged in the intervention. Data were collected in April 2024, with an average time to complete the questionnaire of approximately 20–30 min.

#### 2.3.3 Questionnaire Content and Structure

The questionnaire included two sections. Sec. 1 gathered basic demographic information, including age, gender, level and field of study, urbanicity, and region of permanent residence, prior use of psychological support services, and the use of mental health apps. Sec. 2 consisted of 10 open‐ended prompts designed to elicit in‐depth reflections on participants' perceived changes across four core domains: psychological well‐being, academic and career development, social support attitudes, and interpersonal functioning, and attitudes toward psychological help‐seeking. Participants were invited to describe their thoughts, feelings, and behaviors both before and after participating in the mutual support group. The questions encouraged participants to reflect on whether any changes had occurred and, if so, how the group experience may have contributed to those changes. For example, one question asked: “How would you describe your levels of academic anxiety before participating in the mutual support groups, and how have these levels changed, if at all, after participation? If you experienced any change, how do you think the group contributed to it?”

##### 2.3.3.1 Ethical Considerations

The evaluation was conducted in line with the Department of Psychology's student support protocols and approved by the Faculty of Philosophy Ethical Review Committee at the University of Prishtina in Kosovo, the major public university in Kosovo (Ref. no. 03.40351/1). The procedure adhered to core ethical principles, including informed consent, confidentiality, and voluntary participation.

##### 2.3.3.2 Data Processing

Thematic analysis was conducted following Braun and Clarke's (2006) model, using a deductive approach guided by the predefined focus of the study. The five main thematic domains—(1) Emotional Well‐Being and Stress Relief, (2) Academic Anxiety and Skill Development, (3) Career Confidence and Clarity, (4) Perceptions and Willingness to Seek Help, and (5) Social Support Attitudes and Interpersonal Skills—were pre‐established based on the study's objectives.

A close reading of the data was first conducted to build familiarity with participants' responses. Relevant segments of text were identified and coded in relation to the predefined thematic domains. These codes were then grouped into five domains, and subthemes were developed within each to capture specific and recurring patterns across participant narratives.

All themes and subthemes were subsequently reviewed and refined collaboratively to ensure internal coherence, conceptual clarity, and distinctiveness across the thematic structure. To enhance the credibility and trustworthiness of the analysis, two of the authors of this study independently coded a subset of the data and compared their coding outcomes. Discrepancies were discussed and resolved through consensus among the authors. NVivo and Atlas.ti software were used to support systematic coding, data organization, and cross‐theme comparison.

Finally, narrative summaries were developed for each theme and subtheme, supported by illustrative quotations that reflected both shared and unique participant experiences. These quotations aimed to preserve the authenticity of students' voices and provide concrete insight into the perceived impact of the mutual support groups.

## 3 Results

The findings below are based on students' reflections and are organized into thematic domains. Within each domain, subthemes capture participants' perspectives on their experiences, the changes they reported, and their views on how the mutual support group contributed to shaping these outcomes.

### 3.1 Emotional Well‐Being: Stress, Anxiety, and Mood

#### 3.1.1 Stress Reduction

Many participants recalled feeling overwhelmed by academic and personal demands before joining the mutual support group, often without practical strategies for managing stress. Some noted that they had initially been hesitant to share their struggles, fearing others might not understand or relate. However, discovering that their peers had similar experiences helped normalize these feelings and reduced their sense of isolation. Participants reported that engaging with the group helped ease daily stress, as they learned practical techniques for time management and emotional regulation from one another. Hearing others talk openly about their coping strategies offered emotional reassurance.

After several meetings with the mutual support groups, my stress decreased because I saw that other participants were in a similar situation to me, and they managed to overcome it, which made me think that I could do it too. They also shared some techniques with me, which were very helpful. (First‐year undergraduate student)

#### 3.1.2 Anxiety Alleviation

Several participants reflected on their experiences with social anxiety, particularly in academic contexts such as classroom discussions or group projects. This often stemmed from difficulties in expressing themselves or fear of being judged. The mutual support helped students practice open communication and others' approval. Over time, participants reported feeling calmer and more self‐assured as they developed communication strategies and recognized that others shared similar anxieties. The empathy and encouragement offered by the group were described as key factors in reducing anxiety and building confidence.

Before the group, I used to avoid talking to others because I felt like I would not know what to say or that I would be judged. However, hearing others talk about the same feelings made it easier for me to speak up and feel less anxious. (Second‐year undergraduate student)

#### 3.1.3 Mood Enhancement

Some students reported low mood and emotional flatness before participating in the group, often marked by disconnection and discouragement. Through their involvement, they began to experience moments of optimism and enthusiasm. Feeling heard and accepted within a supportive, nonjudgmental environment fostered emotional balance and a more positive daily outlook.

My mood improved, and I became more enthusiastic after the sessions. I started feeling emotionally lighter, and things didn't seem as hopeless as before. (First‐year undergraduate student)

### 3.2 Academic Functioning: Anxiety and Study Skills

#### 3.2.1 Academic Anxiety Reduction

Before joining the group, several participants reported anxiety related to examination and academic performance. Many avoided discussing these struggles due to the fear of appearing incapable or weak. However, the group setting fostered a shared sense of understanding, as students recognized that others faced similar academic pressures. Over time, participants reported reduced anxiety, particularly regarding test‐taking. This improvement was attributed to learning stress management strategies and receiving emotional support, which helped reframe their perceptions of academic performance.

I always used to get very nervous before tests, to the point that I'd avoid studying. After hearing others share how they deal with this anxiety, I felt less alone and more motivated to face exams. (First‐year master's student)

#### 3.2.2 Improved Study and Time Management Skills

Several students entered the groups without clear study strategies or effective time management habits. Some were unaware of alternative approaches, while others felt hesitant to admit they were struggling. Within the group, a collaborative environment emerged where students shared concrete tools, techniques, and motivational tips, which many described as surprisingly impactful. As a result, participants reported adopting more structured study habits and improved time management. The encouragement and sense of accountability within the group contributed to a greater sense of academic confidence and self‐efficacy.

I learned some techniques to improve concentration and motivation from my group. I applied some of them and noticed the difference in how much I got done. (Second‐year master's student)

### 3.3 Career Confidence and Clarity

Before participating in the group, several students expressed uncertainty about their academic or professional direction. For many, career‐related uncertainty had been a source of ongoing stress that they had rarely shared, often due to fear of judgment or criticism. Within the group setting, open conversations about future plans, employment prospects, and decision‐making helped normalize these concerns. Participants described the experience as both validating and liberating, allowing them to reflect more openly on their paths.

Notably, the group served both those who were unsure about their direction and those who had already made career choices. Students in the first category gained greater clarity and confidence through peer exchange and support. Meanwhile, those with a pre‐existing sense of direction reported feeling more grounded in their decisions after engaging with diverse perspectives.

Before joining the mutual support group, I was unsure about my career path. However, through discussions and shared experiences, I now feel more assured and confident about my decisions. (Third‐year undergraduate student)

I was already confident in my choice, but hearing others talk about their doubts and seeing how they resolved them gave me more courage to stick with mine. (First‐year master's student)

### 3.4 Perceptions and Willingness to Seek Help

#### 3.4.1 Initial Hesitation and Fear of Judgment

At the outset, many participants expressed discomfort with the idea of speaking openly in the group. This hesitation was often driven by fear of being judged or appearing emotionally vulnerable. However, as the sessions progressed and a safe, nonjudgmental environment was established, students reported feeling more at ease. Shared vulnerability played a key role in reducing initial anxieties and fostering trust.

At first, I was nervous about talking—I didn't know if people would judge me. But I quickly saw that others felt the same way, and it helped me speak up. (First‐year undergraduate student)

#### 3.4.2 Shifting Perceptions of Psychological Support

Participants entered the group with varying degrees of stigma or misconceptions about mental health services. Through peer discussion and exposure to others' experiences, these perceptions began to shift. Psychological help has emerged as a normal and healthy resource, rather than a last resort for a crisis. This redefinition emerged through empathy, mutual understanding, and the normalization of conversations about mental health.

I thought that a person who goes to a psychologist is appalling and had no other choice, but after the support groups, I realized that even people who just need to talk can find support from a psychologist. (First‐year undergraduate student)

#### 3.4.3 Increased Openness to Seeking Help

By the end of the sessions, students reported greater willingness to seek professional support in the future. For many, the fear of judgment had subsided, and the idea of seeking help was reframed as an act of self‐care, rather than a sign of weakness. Participants expressed feeling more confident, informed, and open to seeking mental health services.

After the group, I felt much more comfortable with the idea of seeking help. Before, I saw it as something for ‘serious cases.’ Now, I think it is just healthy and normal. (Second‐year master's student)

### 3.5 Social Support Attitudes and Interpersonal Skills

#### 3.5.1 Increased Sense of Belonging and Support

A strengthened sense of connection and mutual care emerged as one of the most consistent outcomes of the support group experience. Students described feeling less isolated and more involved in a supportive peer environment. For many, this translated into boosted confidence in initiating and maintaining social relationships, both within and beyond the group context.

The biggest impact I might have observed from my participation in these groups has been the increase in social support. I perceived that I had more support from others and felt closer to them. (Second‐year master's student)

#### 3.5.2 Improved Communication and Empathy

Many participants reported developing stronger interpersonal skills, including emotional expression and active listening. The group environment allowed them to practice sharing their feelings and responding empathetically to others. This helped dismantle previous communication barriers and encouraged more meaningful emotional connections in everyday life.

Barriers to communication and emotional connection may have been barriers to seeking support. Perhaps I was not sure how to express my feelings or ask others for help. By participating in support groups, I developed new communication and emotional connection skills. (Second‐year undergraduate student)

## 4 Discussion

The findings suggest that peer‐led mutual support groups may contribute to students' emotional well‐being, academic functioning, and interpersonal development. Participants consistently emphasized that connecting with peers facing similar challenges reduced feelings of isolation, normalized distress, and provided new strategies for managing stress and anxiety. These experiences suggest that shared personal narratives have the potential to foster emotional relief and build confidence (Crisp et al. 2020; Byrom 2018), particularly in environments where students may otherwise lack access to consistent psychological support (Hyseni Duraku, Davis, Arënliu, et al. 2024).

In particular, students reported that group discussions contributed to reductions in stress and social anxiety, while also improving their mood and emotional stability. The group setting provides a context where emotions are expressed with empathy and without judgment, fostering a sense of safety and mutual trust. Such outcomes may reflect relational dynamics theorized within resource theory, where the exchange of emotional warmth, validation, and information functions as supportive resources that strengthen resilience and reduce emotional burden (Foa and Foa 1980; Helgeson and Gottlieb 2000).

Academic‐related benefits also emerged, including a reduction in academic anxiety and improvements in time management and study techniques. Students frequently described how peer‐shared strategies and motivational support helped them adopt more effective academic habits and reduce performance pressure. This type of interaction, based on reciprocal support and experiential knowledge‐sharing, aligns with the core tenets of social exchange theory, where mutual benefit and emotional investment are key components in sustaining engagement and fostering personal growth (Stafford 2017). The mutual group setting thus provided an informal yet impactful platform for exchanging practical knowledge and emotional encouragement, which is particularly relevant in formal settings where academic support structures remain underdeveloped (Hyseni Duraku et al. 2023).

Career‐related reflections further suggest that the group format provided students with an emotionally safe space to articulate their uncertainties and receive validation from their peers. For many, hearing others express similar doubts and learning about career opportunities helped reduce internal pressure and foster confidence in their decision‐making. In addition to emotional validation, these discussions demonstrated how mutual support groups can function as informal platforms for peer validation and knowledge exchange—both valuable interpersonal resources that reduce uncertainty and enhance preparedness (Foa and Foa 1980; Coates and Winston 1983; Helgeson and Gottlieb 2000). These dynamics may be significant in settings where institutional career services remain limited (Jemini‐Gashi and Kadriu 2022).

The group experience was also associated with a shift in attitudes toward psychological support. For many, the initial discomfort with expressing vulnerability gave way to greater openness toward mental health conversations. Participants developed a willingness to seek support in the future, in part because the group normalized the act of asking for help. This evolution may be understood through the TPB (Ajzen 1991), which emphasizes how behavioral intentions—such as seeking professional support—can be shaped by changing attitudes, perceived norms, and an increased sense of control. In addition, these attitudinal changes may also reflect the processes described in the ELM (Petty and Cacioppo 1986). Students were not simply passively influenced by others, but often reported thoughtfully engaging with their peers' personal stories, normalizing emotional distress, and reevaluating their own beliefs about help‐seeking. Such peer discussions, rich in personal relevance and emotional depth, may have encouraged central‐route processing, contributing to more enduring changes in attitudes and perceptions. These changes are particularly relevant in the context of widespread stigma and cultural taboos surrounding mental health (Hyseni Duraku, Davis, Arënliu, et al. 2024). The mutual support groups may have helped challenge internalized beliefs—such as the idea that seeking psychological help is only for extreme cases—by offering a space where vulnerability received validation, rather than judgment. Through repeated exposure to open dialog and peer modeling, participants may have experienced a reframing of help‐seeking from a sign of weakness to an act of self‐awareness.

Participants also emphasized the interpersonal benefits of the groups, including improvements in their ability to communicate emotions, listen actively, and develop closer peer relationships. These developments support the idea that mutual support groups may offer benefits beyond emotional relief, helping students build foundational social capacities that contribute to broader personal and academic engagement (Collings et al. 2014; Oarga et al. 2015). In particular, the repeated sharing of experiences and active peer listening contributed to a stronger sense of belonging—an outcome closely tied to student motivation and engagement (Kuh 2003; Axelson and Flick 2010). The findings suggest that peer‐led mutual support groups offer a promising complementary approach to enhancing student well‐being and academic engagement in higher education institutions. Higher education institutions could consider promoting and integrating voluntary peer groups into broader student support services—particularly in formal settings where counseling services or other academic support structures for students remain under development (Jemini‐Gashi and Hoxha 2024; Hyseni Duraku, Davis, Arënliu, et al. 2024). The low‐cost and flexible nature of these groups makes them easy to implement with minimal infrastructure (Mead et al. 2001; Solomon 2004). Small‐scale efforts coordinated by academic units or student‐led initiatives could help create informal spaces for social and emotional support, scholarly exchange, and career reflection. In addition, as students in this study reported increased openness to seeking help, such groups may gradually shift peer norms around mental health and promote help‐seeking behaviors in culturally sensitive contexts (Giamos et al. 2017; Hyseni Duraku et al. 2023, Hyseni Duraku, Davis, Arënliu, et al. 2024). While these findings offer preliminary evidence of the potential of peer‐led mutual support groups, several limitations must be acknowledged, and further research is required to expand on these insights.

First, although the retrospective design was intentionally selected to reduce participant burden and to avoid confronting participants with sensitive topics—due to cultural stigma that could have discouraged group participation—and to minimize response‐shift bias, it remains vulnerable to recall bias, as participants were asked to reflect on their pre‐intervention state from memory. Such self‐assessments may not always accurately reflect baseline experiences.

Second, as a qualitative exploration, the study aimed to capture participants' subjective perceptions through the use of a post‐intervention open‐ended survey. This approach, while practical and less burdensome for participants, may have limited the depth and nuance of responses compared to interviews or focus groups, which allow for follow‐up questions and richer elaboration. Furthermore, the current study did not incorporate objective measures of change, and the absence of a control or comparison group further restricts the possibility of drawing causal conclusions about the intervention's impact. Future studies could strengthen the evidence base by employing experimental or mixed‐methods designs and by integrating more interactive qualitative approaches to capture richer insights into participants' experiences.

Third, the sample was drawn from a single institutional and cultural context, with limited demographic diversity, which limits the transferability of the findings to other settings. Broader samples across different institutions and student populations are needed to capture a more varied range of experiences.

Finally, as data were collected immediately post‐intervention, the study does not provide insight into the long‐term sustainability of the perceived outcomes. Longitudinal research is necessary to explore whether reported improvements in well‐being, coping, and academic attitudes persist over time.

## Conflicts of Interest

The authors declare no conflicts of interest.

## Data Availability

The data that support the findings of this study are available from the corresponding author upon reasonable request.

## References

[pchj70053-bib-0001] Ajzen, I. 1991. “The Theory of Planned Behavior.” Organizational Behavior and Human Decision Processes 50, no. 2: 179–211. 10.1016/0749-5978(91)90020-T.

[pchj70053-bib-0002] Allen, R. , C. Kannangara , M. Vyas , and J. Carson . 2022. “European University Students' Mental Health During COVID‐19: Exploring Attitudes Towards COVID‐19 and Governmental Response.” Current Psychology 42: 20165–20178. 10.1007/s12144-022-02854-0. PMC8831867 35194357

[pchj70053-bib-0003] Alrashidi, O. , H. P. Phan , and B. H. Ngu . 2016. “Academic Engagement: An Overview of Its Definitions, Dimensions, and Major Conceptualisations.” International Education Studies 9, no. 12: 41–52.

[pchj70053-bib-0004] Arënliu, A. , D. Bërxulli , B. Perolli‐Shehu , B. Krasniqi , A. Gola , and F. Hyseni . 2021. “Anxiety and Depression Among Kosovar University Students During the Initial Phase of Outbreak and Lockdown of COVID‐19 Pandemic.” Health Psychology and Behavioral Medicine 9, no. 1: 239–250. 10.1080/21642850.2021.1903327. 34104559 PMC8158185

[pchj70053-bib-0005] Auerbach, R. P. , J. Alonso , W. G. Axinn , et al. 2016. “Mental Disorders Among College Students in the World Health Organization World Mental Health Surveys.” Psychological Medicine 46, no. 14: 2955–2970. 10.1017/S0033291716001665. 27484622 PMC5129654

[pchj70053-bib-0006] Axelson, R. D. , and A. Flick . 2010. “Defining Student Engagement.” Change: The Magazine of Higher Learning 43, no. 1: 38–43. 10.1080/00091383.2011.533096.

[pchj70053-bib-0045] Braun, V. , and V. Clarke . 2006. “Using Thematic Analysis in Psychology.” Qualitative Research in Psychology 3, no. 2: 77–101.

[pchj70053-bib-0007] Byrom, N. 2018. “An Evaluation of a Peer Support Intervention for Student Mental Health.” Journal of Mental Health 27, no. 3: 240–246. 10.1080/09638237.2018.1437605. 29451411

[pchj70053-bib-0008] Casiday, R. , E. Kinsman , C. Fisher , and C. Bambra . 2008. Volunteering and Health: What Impact Does Volunteering Have? Volunteering England. www.volunteering.org.uk/hsc.

[pchj70053-bib-0009] Chang, J. , Y. Yuan , and D. Wang . 2020. “Mental Health Status and Its Influencing Factors Among College Students During the Epidemic of COVID‐19.” Journal of Southern Medical University 40, no. 2: 171–176. 10.12122/j.issn.1673-4254.2020.02.06. 32376528 PMC7086131

[pchj70053-bib-0010] Chen, C. , F. Bian , and Y. Zhu . 2023. “The Relationship Between Social Support and Academic Engagement Among University Students: The Chain Mediating Effects of Life Satisfaction and Academic Motivation.” BMC Public Health 23: 2368. 10.1186/s12889-023-17301-3. 38031093 PMC10688496

[pchj70053-bib-0011] Coates, D. , and T. Winston . 1983. “Counteracting the Deviance of Depression: Peer Support Groups for Victims.” Journal of Social Issues 39, no. 2: 169–194. 10.1111/j.1540-4560.1983.tb00147.x.

[pchj70053-bib-0012] Collings, R. , V. Swanson , and R. Watkins . 2014. “The Impact of Peer Mentoring on Levels of Student Wellbeing, Integration and Retention: A Controlled Comparative Evaluation of Residential Students in UK Higher Education.” Higher Education 68: 927–942. 10.1007/s10734-014-9752-y.

[pchj70053-bib-0013] Corrigan, P. W. , and N. Rüsch . 2002. “Mental Illness Stereotypes and Clinical Care: Do People Avoid Treatment Because of Stigma?” Psychiatric Rehabilitation Skills 6, no. 3: 312–334. 10.1080/10973430208408441.

[pchj70053-bib-0014] Crisp, D. A. , D. Rickwood , B. Martin , and N. Byrom . 2020. “Implementing a Peer Support Program for Improving University Student Wellbeing: The Experience of Program Facilitators.” Australian Journal of Education 64, no. 2: 113–126. 10.1177/0004944120910498.

[pchj70053-bib-0015] Darby Penney, M. L. S. 2018. Defining “Peer Support”: Implications for Policy, Practice, and Research. https://www.ahpnet.com/ahpnet/media/ahpnetmedialibrary/white%20papers/dpenney_defining_peer_support_2018_final.pdf.

[pchj70053-bib-0016] Dearlove, J. , H. Farrell , N. Handa , and C. Pastore . 2007. “The Evolution of Peer Mentoring at the University of Western Sydney.” Journal of the Australian and New Zealand Student Services Association 29: 21–32.

[pchj70053-bib-0017] Długosz, P. 2022. “Mental Health Disorders Among Students From Rural Areas Three Months After Returning to School: A Cross‐Sectional Study Among Polish Students.” Youth 2, no. 3: 271–278. 10.3390/youth2030019.

[pchj70053-bib-0022] Eames, C. , and K. Stewart . 2008. “Personal and Relationship Dimensions of Higher Education Science and Engineering Learning Communities.” Research in Science & Technological Education 26, no. 3: 311–321. 10.1080/02635140802276686.

[pchj70053-bib-0023] Foa, U. G. , and E. B. Foa . 1974. Societal Structures of the Mind. Charles C Thomas.

[pchj70053-bib-0024] Foa, U. G. , and E. B. Foa . 1980. “Resource Theory: Interpersonal Behavior as Exchange.” In Social Exchange: Advances in Theory and Research, edited by K. J. Gergen , M. S. Greenberg , and R. H. Willis , 77–94. Plenum Press.

[pchj70053-bib-0025] Galán‐Muros, V. , J. Roser‐Chinchilla , and N. Hsiung . 2024. Supporting the Mental Health and Well‐Being of Higher Education Students . UNESCO IESALC.

[pchj70053-bib-0026] Geldhof, G. J. , D. A. Warner , J. K. Finders , A. A. Thogmartin , A. Clark , and K. A. Longway . 2018. “Revisiting the Utility of Retrospective Pre‐Post Designs: The Need for Mixed‐Method Pilot Data.” Evaluation and Program Planning 70: 83–89. 10.1016/j.evalprogplan.2018.05.002. 30029016

[pchj70053-bib-0027] Giamos, D. , A. Y. S. Lee , A. Suleiman , H. Stuart , and S. P. Chen . 2017. “Understanding Campus Culture and Student Coping Strategies for Mental Health Issues in Five Canadian Colleges and Universities.” Canadian Journal of Higher Education 47, no. 3: 136–151. 10.7202/1043242ar.

[pchj70053-bib-0028] González, A. , and P. V. Paoloni . 2015. “Behavioral Engagement and Disaffection in School Activities: Exploring a Model of Motivational Facilitators and Performance Outcomes.” Anales de Psicología 31, no. 3: 869–878.

[pchj70053-bib-0029] Helgeson, V. S. , and B. H. Gottlieb . 2000. “Support Groups.” In Social Support Measurement and Intervention: A Guide for Health and Social Scientists, edited by S. Cohen , L. G. Underwood , and B. H. Gottlieb , 221–245. Oxford University Press.

[pchj70053-bib-0046] Howard, G. S. , K. M. Ralph , N. A. Gulanick , S. E. Maxwell , D. Nance , and S. L. Gerber . 1979. “Internal Invalidity in Pretest‐Posttest Self‐Report Evaluations and the Re‐Evaluation of Retrospective Pretests.” Applied Psychological Measurement 3, no. 1: 1–23.

[pchj70053-bib-0030] Hu, S. , and A. C. McCormick . 2012. “An Engagement‐Based Student Typology and Its Relationship to College Outcomes.” Research in Higher Education 53, no. 7: 738–754.

[pchj70053-bib-0018] Hyseni Duraku, Z. 2017. “Factors Influencing Test Anxiety Among University Students.” European Journal of Social & Behavioural Sciences 18, no. 1: 69–78. 10.15405/ejsbs.206.

[pchj70053-bib-0019] Hyseni Duraku, Z. , H. Davis , and E. Hamiti . 2023. “Mental Health, Study Skills, Social Support, and Barriers to Seeking Psychological Help Among University Students: A Call for Mental Health Support in Higher Education.” Frontiers in Public Health 11: 1220614. 10.3389/fpubh.2023.1220614. 37920583 PMC10619655

[pchj70053-bib-0020] Hyseni Duraku, Z. , and L. Hoxha . 2018. “Self‐Esteem, Study Skills, Self‐Concept, Social Support, Psychological Distress, and Coping Mechanism Effects on Test Anxiety and Academic Performance.” Health Psychology Open 5, no. 2: 2055102918799963. 10.1177/2055102918799963. 30225094 PMC6136113

[pchj70053-bib-0047] Hyseni Duraku, Z. 2021. Impact of the COVID‐19 Pandemic on Education and Wellbeing: Implications for Practice and Lessons for the Future. University of Prishtina “Hasan Prishtina” Faculty of Philosophy, Department of Psychology.

[pchj70053-bib-0048] Hyseni Duraku, Z. , H. Davis , A. Arënliu , F. Uka , and V. Behluli . 2024. “Overcoming Mental Health Challenges in Higher Education: A Narrative Review.” Frontiers in Psychology 15. 10.3389/fpsyg.2024.1466060. PMC11670071 39726628

[pchj70053-bib-0049] Hyseni Duraku, Z. , H. Davis , A. Blakaj , A. Ahmedi Seferi , K. Mullaj , and V. Greiçevci . 2024. “Mental Health Awareness, Stigma, and Help‐Seeking Attitudes Among Albanian University Students in the Western Balkans: A Qualitative Study.” Frontiers in Public Health 12. 10.3389/fpubh.2024.1434389 PMC11408363 39296837

[pchj70053-bib-0021] Hyseni Duraku, Z. , J. Konjufca , E. Hamiti , and T. Bajmaku . 2025. “Outcomes of Mutual Support Groups on Students' Mental Well‐Being and Learning: Insights From the COVID‐19 Pandemic.” Health Education. 10.1108/HE-10-2024-0123.

[pchj70053-bib-0031] Jemini‐Gashi, L. , and N. Hoxha . 2024. “Role of Social Support in the Relationship Between Career Self‐Efficacy and Psychological Distress Among Young People During the COVID‐19 Pandemic.” SAGE Open 2024, no. 2. 10.1177/21582440241243172.

[pchj70053-bib-0032] Jemini‐Gashi, L. , and E. Kadriu . 2022. “Exploring the Career Decision‐Making Process During the COVID‐19 Pandemic: Opportunities and Challenges for Young People.” SAGE Open 2022, no. 1. 10.1177/21582440221078856.

[pchj70053-bib-0033] Kuh, G. D. 2003. “What We're Learning About Student Engagement From NSSE: Benchmarks for Effective Educational Practices.” Change: The Magazine of Higher Learning 35, no. 2: 24–32. 10.1080/00091380309604090.

[pchj70053-bib-0034] Mead, S. , D. Hilton , and L. Curtis . 2001. “Peer Support: A Theoretical Perspective.” Psychiatric Rehabilitation Journal 25, no. 2: 134–141. 10.1037/h0095032. 11769979

[pchj70053-bib-0035] Mohammadyari, G. 2012. “Comparative Study of Relationship Between General Perceived Self‐Efficacy and Test Anxiety With Academic Achievement of Male and Female Students.” Procedia—Social and Behavioral Sciences 69: 2119–2123. 10.1016/j.sbspro.2012.12.175.

[pchj70053-bib-0036] Nieuwenhuis, M. , A. S. R. Manstead , and M. J. Easterbrook . 2019. “Accounting for Unequal Access to Higher Education: The Role of Social Identity Factors.” Group Processes & Intergroup Relations 22, no. 3: 371–389. 10.1177/1368430219829824.

[pchj70053-bib-0037] Oarga, C. , O. Stavrova , and D. Fetchenhauer . 2015. “When and Why Is Helping Others Good for Well‐Being? The Role of Belief in Reciprocity and Conformity to Society's Expectations.” European Journal of Social Psychology 45, no. 2: 242–254. 10.1002/ejsp.2092.

[pchj70053-bib-0050] Petty, R. E. , and J. T. Cacioppo . 1986. “The Elaboration Likelihood Model of Persuasion.” In Advances in experimental social psychology, vol. 19, 123–205. Academic Press.

[pchj70053-bib-0039] Pointon‐Haas, J. , L. Waqar , R. Upsher , J. Foster , N. Byrom , and J. Oates . 2024. “A Systematic Review of Peer Support Interventions for Student Mental Health and Well‐Being in Higher Education.” BJPsych Open 10, no. 1: e12. 10.1192/bjo.2023.603. PMC10755562 38098123

[pchj70053-bib-0051] Pratt, C. C. , W. M. McGuigan , and A. R. Katzev . 2000. “Measuring Program Outcomes: Using Retrospective Pretest Methodology.” American Journal of Evaluation 21, no. 3: 341–349.

[pchj70053-bib-0040] Salim, R. M. A. , M. R. Istiasih , N. A. Rumalutur , and D. D. B. Situmorang . 2023. “The Role of Career Decision Self‐Efficacy as a Mediator of Peer Support on Students' Career Adaptability.” Heliyon 9, no. 4: e14911. 10.1016/j.heliyon.2023.e14911. 37025764 PMC10070909

[pchj70053-bib-0041] Solomon, P. 2004. “Peer Support/Peer Provided Services Underlying Processes, Benefits, and Critical Ingredients.” Psychiatric Rehabilitation Journal 27, no. 4: 392–401. 10.2975/27.2004.392.401. 15222150

[pchj70053-bib-0042] Stafford, L. 2017. “Social Exchange Theory: A Cost‐Benefit Approach to Relationships.” In Engaging Theories in Family Communication: Multiple Perspectives, edited by D. O. Braithwaite , E. A. Suter , and K. Floyd , 279–289. Routledge.

[pchj70053-bib-0043] Steinberg, D. M. 2014. A Mutual‐Aid Model for Social Work With Groups. Routledge.

[pchj70053-bib-0044] World Health Organization . 2022. World Mental Health Report: Transforming Mental Health for All . WHO. https://www.who.int/publications/i/item/9789240049338.

